# Supplementary material for: Passive infusion of an S2-Stem broadly neutralizing antibody protects against SARS-CoV-2 infection and lower airway inflammation in rhesus macaques
Source: PLoS Pathog. 2025 Jan 23;21(1):e1012456. doi: 10.1371/journal.ppat.1012456 (PMC11793774; doi:10.1371/journal.ppat.1012456)
Supplement: S1 Text — Supplementary Materials and Methods, Supplementary Figures S1-S10, Supplementary Tables S1-S4, Supplementary References, Table of Supplementary Data Files. (DOCX) [file ppat.1012456.s001.docx]

Supplementary Materials

***LIST OF SUPPLEMENTARY MATERIALS***

Figs A to J

Tables A to D

MDAR Reproducibility Checklist

**Materials and Methods**

**Tissue SARS-CoV-2 RNA Quantification**

Viral load RNA tested via three different sites.

Site 1 Method

For BAL and nasal swabs media placed in RNazol BD (MRC #RB 192) with acetic acid (Sigma Aldrich #CAS64-19-7, RNA was extracted via the RNazol BD protocol. RNA concentration was obtained using the RNA Broad Range Qubit kit (Q10210).  RNA quality was checked via the High Sensitivity RNA Screentape Analysis (Agilent 5067-557) Subgenomic RNA quantification was performed as previously in Corbett et al. 2021(*1*).

Quantitative real-time polymerase chain reaction (PCR) was conducted using TaqMan Fast Virus 1-Step Master Mix (Applied Biosystems) with 5μL template and primers and probes at concentrations of 500nM and 200nM respectively. Reagents for detection of sgRNA_E include a forward primer in the common leader region: sgLeadSARSCoV2_F: 5’-CGATCTCTTGTAGATCTGTTCTC-3’ as well as the following transcript-specific probe and reverse primer: E_Sarbeco_P: 5’-FAM-ACACTAGCCATCCTTACTGCGCTTCG-BHQ1–3’ and E_Sarbeco_R: 5’-ATATTGCAGCAGTACGCACACA-3’. Reagents for detection of sgRNA_N include the aforementioned sgLeadSARSCoV2_F and the following transcript-specific probe and reverse primer: wtN_P: 5’-FAM-TAACCAGAATGGAGAACGCAGTGGG-BHQ1–3’and wtN_R: 5’-GGTGAACCAAGACGCAGTAT-3’. PCRs were performed on a QuantStudio 6 Pro Real-Time PCR System (Applied Biosystems) with reverse transcription at 50°C for 5 min and inactivation at 95°C for 20 sec followed by 40 cycles of PCR at 95°C for 3 sec and 60°C for 30 sec. The limit of detection was 50 copies. Design of RNA standards was previously described(*1*). Final copy numbers of sgRNA per sample were normalized to total RNA concentration of the indicated sample, which had been determined by Qubit following extraction.

Site 2 Method

For nasal swabs placed in 1x DNA/RNA Shield (Zymo Cat#R1200), samples were frozen and sent to Tulane for extraction and quantification. Samples were heat inactivated prior to processing at 56˚C, 30 minutes per Tulane’s Institutional Biosafety stipulations. RNA was extracted from swab samples (nasal, pharyngeal, rectal) using the Zymo Viral RNA Kit (Zymo #R1035) according to the manufacturer's instructions. Viral RNA Buffer (Zymo Catg#R1034) was added at twice the volume of sample in DNA/RNA Shield and swabs were centrifuged in a Zymo IC spin column before removing the swab and adding the remainder of the sample volume. The spin column was washed twice with Wash Buffer before ethanol addition and elution in 50uL DNase/RNase-free water. Samples are stored at -80C until plating and viral load quantification.,

and Bioloanzyer for quality.

 For BAL, 200uL of BAL fluid and cells was added to 2X DNA/RNA Shield (Zymo Cat#R1200) and shipped frozen to Tulane.  Samples were heat inactivated prior to processing at 65 degrees C for 60 minutes per Tulane’s Institutional Biosafety stipulations. RNA is then extracted from bronchoalveolar lavage samples using the Zymo Viral RNA Kit (Zymo #R1035) according to the manufacturer's instructions. Viral RNA Buffer (Zymo Catg#R1034) is added at twice the volume of original sample (200uL sample - 400uL buffer) and sample is then incubated for 30 minutes prior to adding to Zymo IC spin column (Zymo Cat# C1004). Samples are then washed twice with Wash Buffer (Zymo Cat# R1003) before the addition of absolute ethanol. Samples are eluted in 50uL DNase/RNase-free water and stored at -80C until plating and viral load quantification.

Isolated RNA was analyzed in a QuantStudio 6 (Thermo Scientific, USA) using 5uL Sample RNA plated with 1.8uL forward primer, 1.8 reverse primer (Integrated DNA Technologies), 0.5uL probe, 5uL TaqPath 1-Step RT-qPCR (Fisher Cat#A15299), and 5.9uL ddH2O for a total 20uL reaction with the following program: 25°C for 2 minutes, 50°C for 15 minutes, 95°C for 2 minutes followed by 40 cycles of 95°C for 3 seconds and 60°C for 30 seconds. Signals were compared to a standard curve generated using in vitro transcribed RNA of each sequence diluted from 10^8 down to 10 copies. Positive controls consisted of SARS-CoV-2 infected VeroE6 cell lysate. Viral copies per swab were calculated by multiplying mean copies per well by amount in the total swab extract.

Site 3 Methods

**Focus Reduction Neutralization Test (FRNT) Assay**

HeLa-ACE2 cells were seeded in 50µL of complete DMEM at a density of 6x10^4^ cells per well. In a dilution plate, BAL was serially diluted in a 3 ten-fold series and 50µL of diluted BAL was added to the 96-well cell plate. The plate was incubated for 20 hours after which the plate was fixed for 1 hour with 4% paraformaldehyde. The plate was then washed three times with 300µL of 1xPBS/0.05% Tween-20. 25µL of human polyclonal sera diluted 1:500 in Perm/Wash buffer (BD Biosciences 554723) was added to the plate and incubated at room temperature for 2 hours. The plate was washed three times and 25µL of peroxidase goat anti-human Fab (Jackson Scientific, 109-035-006) were diluted 1:1000 in Perm/Wash buffer then added to the plate and incubated at room temperature for 2 hours. The plate was washed three times and 25µL of Perm/Wash buffer was added to the plate and incubated at room temperature for 5 minutes. The Perm/Wash buffer was flicked off and TrueBlue perodixdase substrate was immediately added (Sera Care 5510-0030). Assay was done in triplicate.Modified FRNT assay from Rogers et al. 2020 (46).

**Tissue SARS-CoV-2 RNA Quantification**

This assay was modified from the methods previously described (*2*). Viral RNA was extracted from lung tissue, then amplified and quantified via reserve transcription (RT) qPCR. Lung tissue was collected at day 7 or 8 and placed in 1 mL of TRIzol (Invitrogen Cat#15596018). The samples were homogenized usin a Bead Ruptor 12 (Omni International). The tissue homogenates were centrifuged, and the supernatant was transferred to an RNA purification column (Qiagen). Purified RNA was eluted in 60 µL of DNase-, RNase-, endotoxin-free molecular biology-grade water (Millipore). Purified RNA was then subjected to RT and qPCR with the Centers for Disease Control and Prevention’s N1 (nucleocapside primer sets (forward, 5′-GACCCCAAAATCAGCGAAAT-3′; reverse, 5′-TCTGGTTACTGCCAGTTGAATCTG-3′) and a FAM-labeled probe (5′-FAM-ACCCCGCATTACGTTTGGTGGACC-BHQ1-3′) (Integrated DNA Technologies) on a Bio-Rad CFX96 real-time instrument. For quantification, a standard curve was generated by diluting 2.5 × 10^6 PFU RNA equivalents of SARS-CoV-2. Each run included 11 fivefold serial dilutions of the standard. No-template controls were included for the extraction step as well as the qPCR.

**Tissue Collection and Processing**

Peripheral blood mononuclear cells (PBMCs) were collected from the femoral vein into EDTA tubes with additional serum collected in serum separator tubes. Serum was then processed by the ENPRC’s pathology department for comprehensive blood chemistries. Plasma was collected from the EDTA tubes by spinning at 1,500 x g for 10 minutes at room temperature and then transferring the plasma layer to a 15mL conical and spinning again for another 10 minutes at 600 x g. PBMCs were collected using Sepmate Tubes and the Sepmate protocol with pipetting off the interface instead of pouring (SepMate™-50 Catalog #85450). Following the directed washes, cells were resuspended in ACK lysis buffer (Lonza # 10-548E) and incubated at room temperature for 10 minutes. Samples were then quenched with 2%FBS/DPBS to 50mL and spun again at 300 x g for 10 minutes. Supernatant was removed and samples were resuspended in 2% FBS/DPBS for counting. A 1:1 dilution of 10uL of Trypan blue stain (ThermoFisher # T10282) and 10uL of resuspended cells were added to cell countess slides (Invitrogen # 100078809). Slides were then inserted in the Countess II for determining the percent of live cells and concentration. Cells were then frozen in vials of at least 10 million PBMCs per mL in freezing media containing 10% of DMSO (Millipore Sigma #34869-100mL) in FBS (Gemini # 100-106). 2% FBS/DPBS was created by adding 2% of total volume with FBS (Gemini # 100-106) to DBPS (Corning # 21-031-CM).

Nasopharyngeal swabs were collected under anesthesia using a clean swab (iClean #CYY-96000) that was inserted approximately 2-3 cm deep into the nasal passage. After collection, swabs were placed in either 200uL of DNA/RNA shield (Zymo #R1200-125) or 1mL of DPBS with RNase inhibitor (Invitrogen # AM2694) and then frozen by placing it on dry ice. When processed at a later date, swabs were thawed at room temperature. Once thaw, nasal swabs were vortexed and then squeezed using clean tweezers to remove all media. RNazol (MRC #RB 192) with acetic acid (Sigma Aldrich #CAS64-19-7) was added at a one to one ratio to nasal swab media. Samples were then vigorously vortexed and RNA was extracted using the RNAzol BD protocol.

Swabs placed in 1x DNA/RNA Shield (Zymo Cat#R1200) were frozen and shipped to Tulane for extraction and processing.

To collect non necropsy BAL, a 14Fr, sterile, single-use pediatric suction catheter (Covidien #37424) was inserted into the trachea and directed into a mainstem bronchi. The catheter was advanced until it was secured into a distal subsegmental bronchus. 35-70 mL of physiological saline was instilled into the bronchus and then manually aspirated back into a syringe to obtain a minimum of 20 mL of lavage fluid and placed into a 50mL conical. If less than 20mL was recovered, an additional 10-15mL was administered again and a second attempt was taken and placed into a second 50mL conical. BAL was then filtered through a 70uM strainer (Falcon #352350). 1mL of BAL fluid and cells were taken to perform viral load testing by adding RNazol and Acetic Acid into a 5mL microcentrifuge tube. After vigorous vortexing, samples were frozen for later RNA extraction using the RNAzol BD kit. After viral loads were taken, BAL was spun at 300 x g for 10 minutes to pellet the cells. BAL supernatant was collected for MSD and plaque assay. BAL cells were then lysed with the ACK lysis buffer for 10 minutes at 37˚C and the reaction was quenched using DPBS. Cells were then spun and counted in the same manner as PBMCs. At least 100,000 cells were set aside for 10x sequence and the remaining cells were divided between the isotype control and stained panel. At necropsy, an additional 100mL of Post Mortem BAL fluid was taken by placing a large sterile irrigation syringe with the plunger removed into the lungs. Approximately 150 mL of sterile 1 x PBS is poured into the lungs via the syringe to infuse both sides. The syringe is then removed and plunger replaced. The syringe goes back into the trachea opening and the lavage fluid is pulled back and dispensed into 50mL conicals. Post mortem BAL sample is processed the same way as non-necropsy through a 70uM filter and lysing. After samples for viral load, supernatant and 10x were taken for pre-mortem samples, the cells for pre and post mortem were combined together for staining. At necropsy, at least 5 million cells were stained for both the isotype and staining panel with any remaining frozen down using the same freezing media as PBMCs.

At necropsy, the tissues above were collected as well as right caudal lung, spleen, and hilar lymph nodes.

Caudal lung is processed by following the steps indicated in Viox et al 2023 (5) with the following changes. During the 1 hour 37˚C incubation, samples were placed on a 50mL tube rotator at 10 rpm (Thermo Scientific # 888810001). Samples were run at least 6 times on the pre-loaded M_Lung_02_01 to ensure proper blending of the tissue. After, the tissue is strained over 100uM filters (Falcon # 352360) and the digestion reaction is quenched with R10. Cells are then centrifuged at 1100 x g for 10 minutes. The supernatant is removed and cells are washed again with R10 and centrifuged at 1100 x g for 10 minutes. The cells are lysed using 10mL of ACK lysis and incubated at 37˚C for 10 minutes. Lysing was then quenched using DPBS and cells were spun down at 1100 x g for 10 minutes. Cells were then counted using the same manner as PBMCs and aliquoted for 10x sequencing, bulk RNA sequencing and freezing.

Cells were used for 10x sequencing and the extra were saved in the same manner as PBMCs.

Spleen was processed in a similar manner to the lung but without the digestion step. Spleen was dissected using blunt scissors and added to C tubes with 5mL of R10. Using the same M_Lung 02_01 protocol run at least 6 times, spleen was blended then passed through a 100uM filter. Due to the nature of the sample, it is then passed through a 70uM filter and then 40uM filter (Falcon #352340) to remove the debris. Cells were then washed using R10 and then spun at 750 x g for 10 minutes to pellet. Supernatant was removed and the cells were washed with DPBS then centrifuged at 750 x g for 10 minutes. Cells were lysed with 20mL of ACK for 10 minutes. The lysing reaction was quenched with DPBS and cells were pelleted following the same speed and time. Cells were counted and frozen for later use.

Lymph nodes were processed by first trimming the fat from the tissue and placing it into a C tube with DBPS. In the gentleMACs dissociator on the preloaded program M-Brain 01_01, lymph nodes were blended. The blended tissue is then strained over a 70uM filter into a 50mL conical and rinsed with DPBS. Cells were centrifuged at 300 x g for 10 minutes at room temperature. After the supernatant is removed, cells are lysed with ACK lysing buffer and incubated at room temperature for 10 minutes. The reaction is stopped by topping off with DPBS and cells are then centrifuged at 300 x g for 10 minutes. Cells are then counted and aliquoted for freezing vials of 10M cells per 1mL of freezing media described above.

In addition to extracting mononuclear cells from lymph nodes, lung and spleen, cassettes of tissues fixed in 4% paraformaldehyde were taken. Following removal, tissues were placed into jars of 4% PFA and fixed for at least 24 hours. Tissues were then cut and placed into cassettes which were then placed in a fresh jar of 4% PFA for additional fixing.

**ARTIC Library Generation**

Selected RNA samples were converted into cDNA following the manufacturer instructions in the SuperScript™ IV First-Strand Synthesis System kit (Invitrogen, 18091200). The obtained cDNA was amplified using the ARTIC V4.1 nCOV-2019 Amplicon Panel kit (Integrated DNA Technologies (IDT), 10011442) developed by the ARTIC Network and consisted of 98 primers within two pools to detect presence of mutations within known variants of interest or newly emerging variants(*3*). Two separate PCR reactions were performed on small aliquots of cDNA to ensure that each sample was amplified with both ARTIC primer pools required for overlapping size distribution. Both PCR reactions consisted of 5.75 μL of Q5® High-Fidelity DNA Polymerase (New England Biolabs, M0491L) 3.6 μL of the respective ARTIC Primer Pool 1 or 2 (10 μM), 6 μL cDNA and 9.65 μL of Nuclease free water for a total reaction volume of 25 μL and cycling conditions of 1× (98 °C, 30s), 35× (95 °C, 15 s; 63 °C, 5 min). The amplified cDNA products were merged for each sample and submitted to purification of 0.8X SPRISelect size selection bead cleanup (Beckman Coulter, B23318). The xGen™ DNA Library Prep EZ kit (IDT, 10009821) was used to prepare next-generation sequencing (NGS) libraries following the protocol IDT provided with ≤100 ng input of ARTIC amplified cDNA, 14 minutes fragmentation time, 5 indexing PCR cycles, and dual indexing with xGen™ UDI 10nt Primer Plates 1-4 (IDT, 10008052). Sample concentration and quality were measured using FilterMax F3 (Molecular Devices, LLC) with Qubit™ 1X dsDNA High Sensitivity solution (Invitrogen, Q33231) and 4200 Tapestation System (Agilent, G2991BA) with D5000 DNA ScreenTapes and reagents (Agilent, 5067-5588). NGS libraries were sequenced on a NovaSeq6000 SP flow cell system (Illumina, Inc) using 150 paired-end sequencing, targeting 1 million reads per sample.

**Expression and purification of monoclonal antibodies CC40.8 and PGT121**

Monocloncal antibody expression and purification was conducted as previously described (*4*). Plasmids of the variable heavy and light chains of CC40.8 and PGT121 were generated in IgG1 and expressed in Expi293F cells. 228µg heavy chain plasmid and 572µg of light chain plasmid were added into 100µL of Opti-MEM (Thermo Fisher Scientific, catalog #31985070), after filtering with 0.22µM Steriflip (Millipore, catalog #SCGP00525), 800µL of FectoPro (Polyplus, catalog #116-001) reagent was added into the mixture and inverted. After incubating at room temperature for 10 minutes, the mixture was added to 900mL of Expi293F cells at a cell density of 2.8-3.0e6 cells/mL and incubated in a shaker with 80% humidity and 5% CO_2_. After 24hr, 10mL of 0.3M Valproic acid and 9mL of 45% glucose were added to the cell culture. Five days post transfection, Expi293F supernatant was harvested by centrifugation at 1250xg for 15 minutes before filtering with a 0.22µM membrane filter. Protein A Sepharose (GE Healthcare Cat# 17096302) was added to the supernatant and was rotated overnight at 4°C overnight. The solution was then loaded into Econo-Pac columns (BioRad Cat# 7321010), washed with 3 column volume of PBS, and antibodies were eluted with 15mL of 0.2 M citric acid (pH 2.67). The elution was neutralized with 4.5mL of 2M Tris Base solution prior to buffer exchanging into PBS with dialysis cassettes. 30K Amicon centrifugal filters (Millipore Cat# UFC903024) were used to concentrate the antibodies into smaller volumes.

**Anti-Spike Antibody Detection in BAL Supernatant and Serum Samples by ELISA**

BAL Supernatant and serum samples were obtained on day -60-30, -4, 0, 2, and 7 or 8 to quantify CC40.8 titers. Spike protein diluted to 2µg/mL in 1xPBS was coated on ELISA plates overnight at 4°C and then washed three times with 100µL of 1xPBS/0.05% Tween-20. After blocking the plates with 50µL of 3%BSA/1xPBS for 1 hour at room temperature, 12.5µL the NHP serum dilution series and CC40.8 dilution series for a standard curve were added to the plate and incubated for 1 hour at room temperature. Plates were wash three times with 100µL of 1xPBS/0.05% Tween-20 before adding 12.5µL of alkaline phosphatase (AP)-conjugated goat anti-human IgG Fc secondary antibody (Jackson ImmunoResearch, catalog #109-055-008) diluted in 1%BSA/1xPBS. After washing the plates three times with 100µL of 1xPBS/0.05% Tween-20, 12.5µL of AP substrate was added for detection. Plates were then read at 405 nm, and the data was analyzed with Graphpad Prism 9. Serum samples were ran in triplicate.

**Single-cell RNA-Seq bioinformatic analysis of BAL and Lung cells**

The cellranger v6.1.0 (10X Genomics) pipeline was used for processing the 10X sequencing data and the downstream analysis was performed using the Seurat v4.0.4 R package. A composite reference comprising of Mmul10 from Ensembl release 100 and SARS-CoV2 (strain MT246667.1 - NCBI) was used for alignment with cellranger. The percentage of SARS-CoV-2 reads was determined using the PercentageFeatureSet for SARS-CoV2 genes. For BAL samples, a total of 107,830 cells across all animals passed quality control (QC) and were used for analyses. For lung samples, a total of 101,766 cells passed upstream QC and were used for analysis. The bioinformatic processing of scRNA-Seq data and subsequent analysis was performed as described previously for BAL samples (*5*) and lung samples (*6*). For single-cell RNA-Seq, approximately 20,000 cells were loaded onto the 10X Genomics Chromium Controller in the BSL3 facility using the Chromium NextGEM Single Cell 5’ Library & Gel Bead kit according to

manufacturer instructions (*7*).

For BAL samples, the samples were demultiplexed using HTODemux function in Seurat, The gene expression matrix was filtered to include protein coding genes and exclude genes encoded on Y chromosome, B and T cell receptor genes, mitochondrial genes, RPS and RPL genes and SARS-CoV2 genes. The cells were further filtered on the following criteria: nFeature_RNA >=500 and <= 3500, ncount_RNA >=250 and log10GenesPerUMI > 0.8. After filtering, the samples were normalized using SCTransform method (*7*) and integrated using the first 30 dimensions with the default CCA method (*8*). Two samples were dropped - two due to low cell numbers (RZn18 2 dpi and ROk18 2 dpi). The integrated object was split into individual samples and after filtering the three samples, the remaining samples were normalized using the SCTransform method (*7*) and then integrated using the reciprocal PCA method (*8*). The first 30 dimensions were used with the FindIntegrationAnchors, FindUMAP and FindNeighbors method. Clustering was carried out using the default Louvain method and the resolution was set to 1. Cell annotations were carried out based on the expression of canonical markers in seurat clusters and SingleR v1.4.0 library (Blueprint Encode database) (*9*) annotations were used as a guide. As a distinct cluster could not be determined for neutrophils based on the expression of canonical marker genes, the SingleR annotations were used for neutrophils. Differential gene expression analysis was carried out using the FindMarkers function with “MAST” (*10*) method. To further classify the macrophages/monocytes in BAL, only cells in the largest cluster comprising the macrophages/monocytes were further processed. The subset function was used to get these cells followed by splitting the object in individual samples. Downstream processing was performed as previously described (*5*).

For lung samples, we processed sections of lower (caudal) lung obtained from animals necropsied at 7 or 8 dpi (n=3 PGT121, n=3 10mg/kg, n=3 0.1 mg/kg). The cellranger pipeline was used as described above and filtered counts were read into Seurat using the Read10X_h5 function. QC filtering was performed as described above. The first 30 dimensions were used and clustering was carried out with the resolution set to 0.1 using the default Louvain algorithm in seurat. The clusters were annotated based on the expression of canonical markers and roughly divided into four major subsets: epithelial, myeloid, lymphoid and others. Each subset was then clustered separately to fine tune the cell type annotations. The human Lung v1 reference (*10*) in Azimuth (*11*) was used to guide the cell annotations. Based on the expression of canonical markers, some clusters were classified as doublets and some remained unassigned. After removing the doublets and unassigned clusters, UMAPs showed some additional cells that coincided with the removed doublets/unassigned clusters, and these were removed as well. Finally, a total of 101,766 cells were used subsequently for downstream analysis. Differential gene expression analysis was carried out using the FindMarkers function with “MAST” method . Over –representation analysis was carried out using clusterProfiler v4.5.0.992 (*12*) with Hallmark, Reactome, KEGG and BioCarta genesets from the msigdb database (*130-134*). The msigdbr v7.5.1 library (https://igordot.github.io/msigdbr/) was used for retrieving the msigdb databases. Downstream processing was performed as previously described (*5*).

**Macrophage Flow Cytometry Immunophenotyping**

Multi–parameter flow cytometric analysis was performed on fresh mononuclear cells isolated from BAL using the following mAbs: anti-CD45 BUV805 (clone D058-1283; 2.5uL; cat # 742055); anti-CD163 BUV395 (clone MAC2-158; 2.5uL; cat # 568191); anti-CD14 BUV786 (clone M5E2; 7.5uL; cat # 563698); and anti-CD206 BV480 (clone 19.2; 7.5uL; cat # 746279) all from BD Bioscience; anti-CD16 PE-Cy5 (clone 3G8; 20uL; cat # 302010) from Biolegend; Fixable Viability Dye eFluor780 4x (Cat. No.: 65-0865-14; 25uL) from eBioscience. After 10x captures and sample banking, all remaining BAL cells were split into 3 aliquots (one for unstained cell control, one for the flow panel, and one for compensation controls). Cells set aside for compensation controls from all animals sampled on the same day were pooled and then split evenly into enough tubes to make cell-based compensation controls for each antibody in the panel. For unstained cells, 2mL of stain buffer, added then spin down at 800 x g at RT for 10 minutes, then resuspend in 100uL of 4% PFA for 10 minutes. Then 300uL of stain buffer were added to bring unstained cells to 1% PFA. Unstained samples were kept at 4˚C until ready to run flow. For the stained samples and compensation controls, 100uL of FC block were added per sample (5uL of stock and 95uL of stain buffer), then incubated in dark at RT for 30 minutes. After samples were washed and spun again, 100uL of staining master mix or singular antibody for compensation control were added and incubated for 30 min in the dark at RT, followed by another wash and spin. Samples were then resuspended in 100uL of 4% PFA for 10 minutes, and then brought to 300uL of stain buffer for a final concentration of 1% PFA. Samples were kept at 4˚C until ready to run. Due to the high autofluorescence of alveolar macrophages, BAL cells were used as the compensation controls instead of beads and fluorophores were selected for channels with minimum autofluorescence, with unstained BAL cells spiked in to each compensation control. Macrophages were gated on singlets, CD45+, FSC and SSC characteristic of granulocytes and alveolar macrophages, live cells, CD14+, and CD163+ populations, and assessed for MRC1 expression. Due to the high variability of the autofluorescence commonly observed in airway macrophage populations, we found it necessary to gate for CD163 and MRC1 using each animal’s pre-infection BAL sample, and to then apply these gates to the later timepoints for that same animal. Samples were run on BD FACSymphony A5 driven by FACS DiVa software and analyzed with FlowJo (Version 10.10).

**Lung Histology**

Lung samples from nonhuman primates were fixed in 10% neutral buffered formalin, processed, and blocked in paraffin for histological analysis. All samples were sectioned at 5 μm and stained with hematoxylin-eosin (H&E) for routine histopathology. Pathology was scored as described: **(0)**: inflammation minimal to absent; **(.5)**: minimal to mild inflammation (very mild alveolar capillary hypercellularity), areas of perivascular and/or peribronchial (PBr) / peribronchiolar (Pbr) inflammation are rare to absent; **(1)**: Mild to moderate inflammation (alveolar hypercellularity with occasional cells within alveolar spaces), noticeable interstitial capillary expansion, and occasional perivascular (PV) and/or PBr/br infiltrates; **(2)**: Moderate to severe inflammation; alveolar hypercellularity, moderate numbers of cells in alveolar spaces, regular PV and PBr/Pbr infiltrates, occasional areas of diminished airspace in section; *for samples with 25-50% tissue affected, an additional "+/- will be factored in to score*; **(3)** = Severe inflammation; prominent PV/PBr/Pbr inflammation, substantially diminished air spaces (inflammation/consolidation) +/- edema; *for samples with <50% of the tissue affected, an additional "+" will be factored into score.*

**Figures**

| 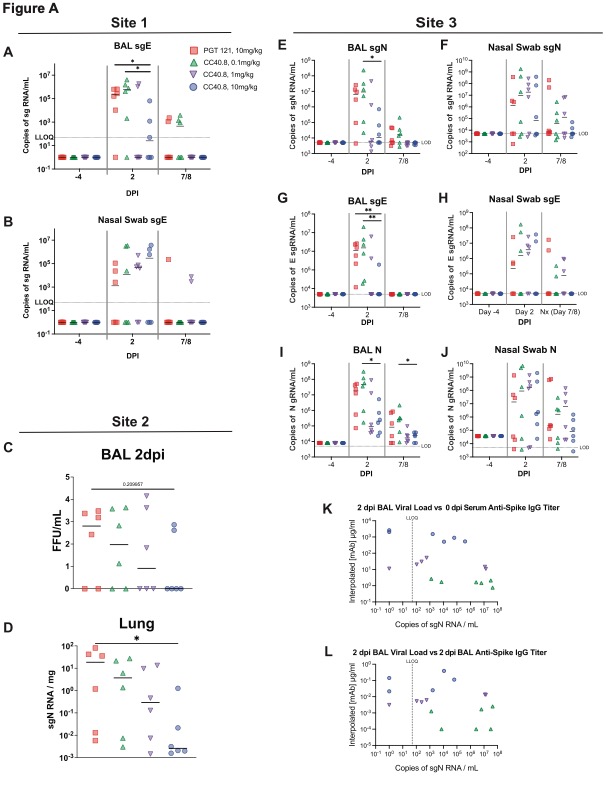 |
| --- |
| **Fig A. CC40.8 reduced BAL and lung but not nasopharyngeal viral loads in SARS-CoV-2-challenged RMs.** Viral sgRNA N and sgRNA E quantification was replicated by two independent laboratories (Site 1 and Site 3). Viral gRNA quantification was performed by site 3. Tissue viral loads and viral plaque assays were performed by site 2. (**A** to **B**) Viral loads based on qPCR analysis generated by site 1. sgRNA-E viral loads for BAL (A) and nasal swab (**B**). (**C** to **D**) Viral loads generated by site 2. **(C**) SARS-CoV-2 infectious virus titers (PFU) as determined by plaque assay from lung tissue at day 5 after infection. (**D**) SARS-CoV-2 viral RNA loads based on the qPCR analysis of lung tissue at day 5 after infection. (**E** to **J**) Viral loads based on qPCR analysis generated by site 3. (**E**) sgRNA-N viral loads for BAL. (**F**) sgRNA-N viral loads for nasopharyngeal swabs. (**G**) sgRNA-E viral loads for BAL. (**H**) sgRNA-E viral loads for nasopharyngeal swabs. (**I**) gRNA-N viral loads for BAL. (**J**) gRNA-N viral loads for nasopharyngeal swabs. (**K**) Correlation of SARS-CoV-2 sgRNA-N levels in the BAL with serum levels of anti-spike IgG titers. (**L**) Correlation of SARS-CoV-2 sgRNA-N levels in the BAL with BAL levels of anti-spike IgG titers. Control PGT121-treated RMs are depicted with red squares, CC40.8 0.1mg/kg-treated RMs depicted with green upward pointing triangles, CC40.8 1mg/kg-treated RMs depicted with purple downward pointing triangles, CC40.8 10mg/kg-treated RMs depicted with blue circles. Black lines represent the median titer in RMs from each respective treatment group. Statistical analyses were performed using two-sided nonparametric Mann-Whitney tests. **P* < 0.05. |

| 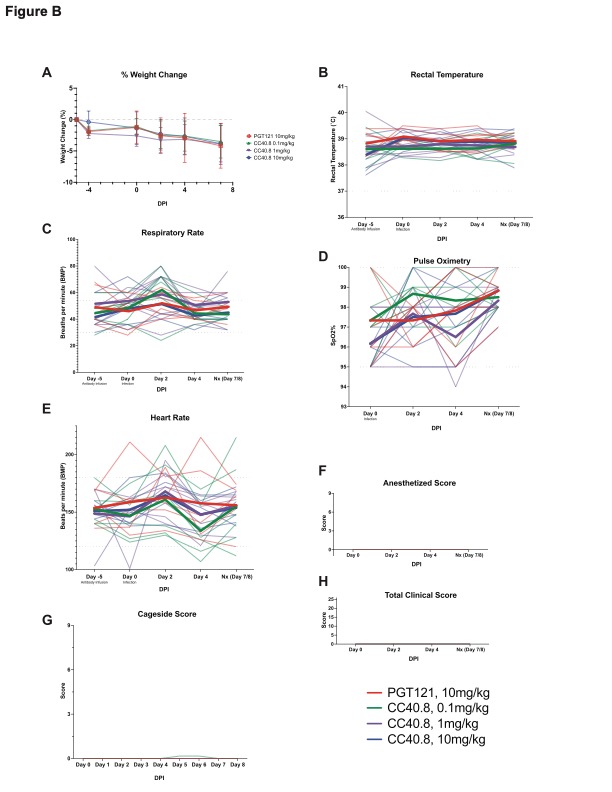 |
| --- |
| **Fig B. Administration of CC40.8 mAb was safe and well-tolerated in RMs**. (**A** to **E**) Longitudinal measurements of (**A**) percent weight change from preinfection baseline, (**B**) rectal temperature, (**C**) respiratory rate, (**D**) pulse oximetry, and (**E**) heart rate in RMs. Statistical analysis between timepoints was performed using two-sided Wilcoxon matched-pairs signed rank tests. * p-value < 0.05. (**F** to **H**) Anesthetized scores, cage-side scores, and total clinical scores of PGT121 and CC40.8-treated SARS-CoV-2-infected RMs. Statistical analyses for panels b-d were performed using non-parametric Mann-Whitney tests. * p-value < 0.05, Black dotted horizontal lines indicate normal ranges for measured parameters for adult indoor RMs. Bolded red, green, purple and blue lines indicate averages for PGT121, CC40.8 0.1mg/kg, CC40.8 1mg/kg, and CC40.8 10mg/kg-treated RMs respectively. |

| 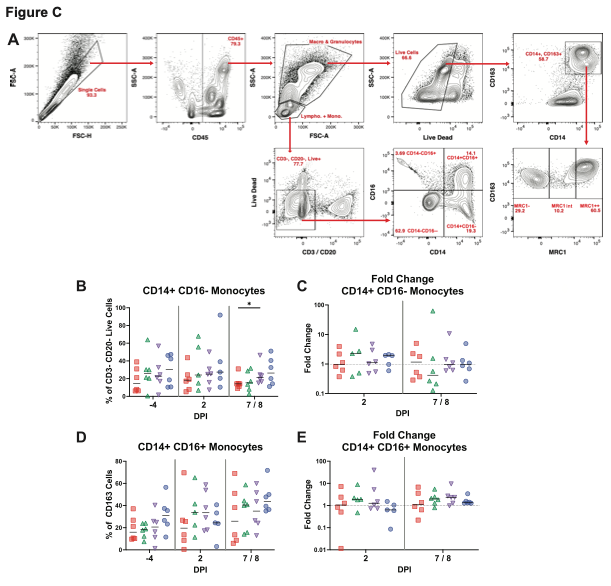 |
| --- |
| **Fig C. Flow gating strategy for macrophage and monocyte levels in BAL of RMs.** (**A**) Gating strategy for innate immune cell phenotyping panel in BAL (shown in Fig 3). (**B**) Frequency of CD14+CD16- monocytes in BAL mononuclear cells and (**C**) fold change relative to -4 dpi. (**D**) Frequency of CD14+CD16+ monocytes in BAL mononuclear cells and (**E**) fold change relative to -4 dpi. Control PGT121-treated RMs are depicted with red squares, CC40.8 0.1mg/kg-treated RMs depicted with green upward pointing triangles, CC40.8 1mg/kg-treated RMs depicted with purple downward pointing triangles, CC40.8 10mg/kg-treated RMs depicted with blue circles. Statistical analyses were performed using two-sided non-parametric Mann- Whitney tests. * p-value < 0.05 |

|  |
| --- |
| **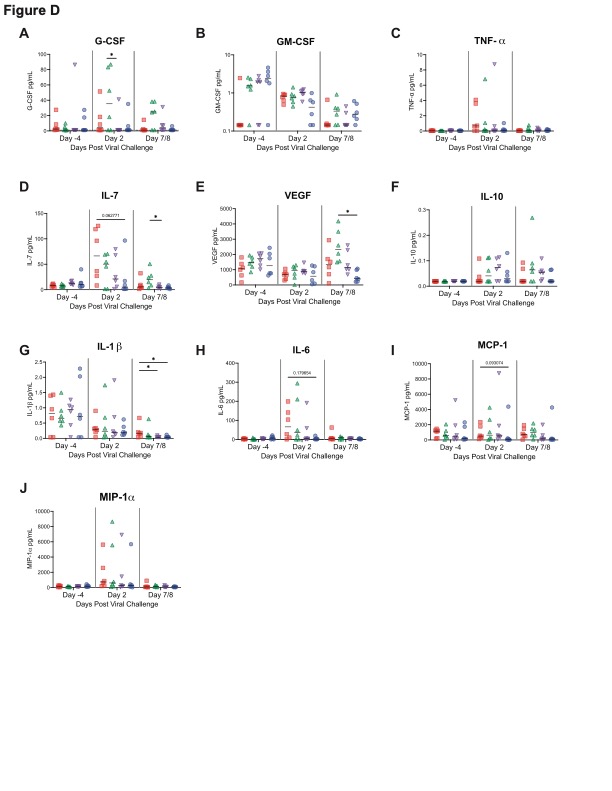**  **Fig D. Cytokine and chemokine levels in BAL of RMs**. (**A** to **J**) Levels of cytokines and chemokines (pg/mL) in the BAL of RMs at -4, 2 and 7/8 dpi. Control PGT121-treated RMs are depicted with red squares, CC40.8 0.1mg/kg-treated RMs depicted with green upward pointing triangles, CC40.8 1mg/kg-treated RMs depicted with purple downward pointing triangles, CC40.8 10mg/kg-treated RMs depicted with blue circles. Black lines represent the median level in RMs from each respective treatment group. Statistical analyses were performed using two-sided nonparametric Mann-Whitney tests. **P* < 0.05. |

|  |
| --- |
| ****  **Fig E. Expression of marker genes in BAL single cells.** (**A**) UMAP of BAL samples colored by clusters determined using Seurat and annotated cell types. (**B**) Dot Plot showing expression of canonical marker genes in Seurat clusters. (**C**) Dot Plot showing expression of canonical marker genes in annotated cell types. |

|  |
| --- |
| **Fig F. Effect of CC40.8 treatment on BAL single cells following SARS-CoV-2 challenge.** (**A**) UMAP split by time point and treatment showing BAL macrophages/monocytes mapped to the reference macrophage/monocytes from lungs of healthy RM. (**B**) Violin plots showing the percentage of viral reads in different BAL cell subsets from all RMs at 2 dpi. Dot plots showing the expression of selected ISGs, inflammatory genes, chemokines, and inflammasome genes in CD163+ MRC1+ and CD163+ MRC1+ TREM2+ macrophages. The size of the dot indicates the percentage of cells that express a given gene, and the color indicates the level of expression. **(D-G)** Correlation of single-cell macrophage and monocyte subsets with SARS-CoV-2 titers (sgRNA-N) within the BAL at 2 dpi for (**D**) CD16+ monocytes, (**E**) CD163+MRC1- macrophages, (**F**) CD163+MRC1+ TREM2+ macrophages, and (**G**) CD163+MRC1- macrophages. |

|  |
| --- |
| **Fig G. Annotation of lung cells.** (**A** to **D**) UMAPs displaying Seurat clustering and cell-type annotations based on the expression of marker genes and dot plots with canonical marker genes for (**A**) epithelial, (**B**) lymphoid, (**C**) myeloid, and (**D**) other (stromal and endothelial) subsets. For each category, cells were clustered separately following preliminary cell-type annotation. The size of the dot in the dotplots indicates the percentage of cells that express a given gene, and the color indicates the level of expression. |

|  |
| --- |
| **Fig H. Effect of CC40.8 treatment on gene expression in lung cells** (**A** to **D**) Dot plots showing gene expression in lung cells from PGT121, CC40.8 0.1 mg/kg, and CC40.8 10mg/kg-treated RMs. (**A**) Expression of chemokines in lung cells at 7/8 dpi. (**B**) Expression of inflammasome genes in lung cells at 7/8 dpi. (**C**) Expression of genes related to programmed cell death in lung cells at 7/8 dpi. (**D**) Expression of genes related to inflammation in lung cells at 7/8 dpi. The size of the dot indicates the percentage of cells that express a given gene, and the color indicates the level of expression. |

|  |
| --- |
| **Fig I. SARS-CoV-2 iSNV position and frequency** (**A** to **H**) Plots of iSNV frequency across the genome for each experimental group at 2 dpi (**A, C, E, G**) and 7/8 dpi **(B, D, F, H**). Control PGT121-treated RMs are depicted in red (**A** to **B**), CC40.8 0.1mg/kg-treated RMs depicted in green (**C** to **D**), CC40.8 1mg/kg-treated RMs depicted in purple (**E** to **F**), CC40.8 10mg/kg-treated RMs depicted in blue (**G** to **H**). SARS-CoV-2 iSNV isolated from BAL supernatant were called against the input consensus sequence with iVar v1.3.1, setting the maximum depth at 29 million bases, minimum quality score at 15 and minimum frequency at 1%, and were only considered if present in both replicate libraries. |

|  | |
| --- | --- |
| **Fig J. H&E staining of RM Caudal Lung** (**A** to **C**) Photomicrographs of caudal lung tissue sections of control (**A**) and CC40.8 10mg/kg treated (**B**) nonhuman primate groups at 7 or 8 dpi. All photomicrographs taken at either 10x or 20x magnification (scale bars represent 100µm and 50µm, respectively). Average lobe pathology scores for each animal (**C**). Control PGT121-treated RMs are depicted with red squares, CC40.8 0.1mg/kg-treated RMs depicted with green upward pointing triangles, CC40.8 1mg/kg-treated RMs depicted with purple downward pointing triangles, CC40.8 10mg/kg-treated RMs depicted with blue circles. Statistical analyses were performed using two-sided nonparametric Mann-Whitney tests.  . | |
|  |  |
| **Table A.** **Antibody Infused RM characteristics.** Animal ID. Sex. Age in months relative to -5 dpi and weight taken from beginning of study in kg. Antibody treatment and collection group. Day post infection that necropsies were performed. Baseline Anti-Spike Antibody titers as measured with SARS-CoV-2 spike binding ELISA, in area under the curve calculations, including positive (RLp17) and negative (RAg5) controls. Curves were generated using absorbance (OD405nm) values plotted against log-transformed serum concentrations. Baselines were corrected based on ELISA absorbance background values. Positive control serum (RLp17) was sampled from a RM infected with WA.1 SARS-CoV-2 at 21 dpi and negative control serum (RAg5) was sampled from a RM prior to the COVID-19 pandemic (5/29/2014). |  |

|  |
| --- |
| **Table B. Coronavirus Vaccine and Treatment Evaluation Network (CoVTEN) standard clinical assessment for cage-side scores, related to Fig B.** Cage-side scores were performed at 0, 1, 2, 3, 4, 5, and 7 dpi and added to anesthetized scores to obtain the total clinical score for each dpi. Cageside scores were based on responsiveness, discharges, respiratory rate, respiratory effect, cough, and fecal consistency and were completed prior to anesthesia. |

|  |
| --- |
| **Table C. Coronavirus Vaccine and Treatment Evaluation Network (CoVTEN) standard clinical assessment for anesthetized scores, related to Fig B.** Anesthetized scores were performed at 0, 1, 2, 3, 4, 5, and 7 dpi and added to cageside scores to obtain the total clinical score for each dpi. Anesthetized scores were based on discharges, respiratory character, and hydration. Body weights (kg), body condition scores, respiratory rates (bpm), SpO2 (%), and rectal temperatures (°F) were also recorded during anesthetic accesses. |

|  |
| --- |
| **Table D. Correlation of cytokine and chemokine levels with SARS-CoV-2 titers within the BAL at 2dpi.** Cytokines and chemokines levels (pg/mL) in BAL fluid relative to −4 dpi measured by MSD immunoassay. SARS-CoV-2 titers were measured in BAL via detection of (sgRNA-N) by qPCR. Grey shaded rows indicate significant correlation. |

***REFERENCES***

1. Corbett KS, Nason MC, Flach B, Gagne M, O’Connell S, Johnston TS, et al. Immune correlates of protection by mRNA-1273 vaccine against SARS-CoV-2 in nonhuman primates. Science. 2021;373(6561):eabj0299.

2. Zhou P, Yuan M, Song G, Beutler N, Shaabani N, Huang D, et al. A human antibody reveals a conserved site on beta-coronavirus spike proteins and confers protection against SARS-CoV-2 infection. Sci Transl Med. 2022;14(637):eabi9215.

3. Integrated DNA Technologies I. ARTIC SARS-CoV-2 Amplicon Panel; 10011442 2024 [Available from: https://www.idtdna.com/pages/products/next-generation-sequencing/workflow/xgen-ngs-amplicon-sequencing/predesigned-amplicon-panels/artic-sc2-amp-panel.

4. Zhou Y, Zhi H, Teng Y. The outbreak of SARS‐CoV‐2 Omicron lineages, immune escape, and vaccine effectivity. J Méd Virol. 2023;95(1):10.1002/jmv.28138.

5. Upadhyay AA, Viox EG, Hoang TN, Boddapati AK, Pino M, Lee MY, et al. TREM2(+) and interstitial-like macrophages orchestrate airway inflammation in SARS-CoV-2 infection in rhesus macaques. Nat Commun. 2023;14(1):1914.

6. Esaulova E, Das S, Singh DK, Choreño-Parra JA, Swain A, Arthur L, et al. The immune landscape in tuberculosis reveals populations linked to disease and latency. Cell Host Microbe. 2021;29(2):165-78.

7. Zheng GXY, Terry JM, Belgrader P, Ryvkin P, Bent ZW, Wilson R, et al. Massively parallel digital transcriptional profiling of single cells. Nat Commun. 2017;8(1):14049.

8. Hafemeister C, Satija R. Normalization and variance stabilization of single-cell RNA-seq data using regularized negative binomial regression. Genome Biology. 2019;20(1):296.

9. Stuart T, Butler A, Hoffman P, Hafemeister C, Papalexi E, Mauck WM, et al. Comprehensive Integration of Single-Cell Data. Cell. 2019;177(7):1888-902.e21.

10. Finak G, McDavid A, Yajima M, Deng J, Gersuk V, Shalek AK, et al. MAST: a flexible statistical framework for assessing transcriptional changes and characterizing heterogeneity in single-cell RNA sequencing data. Genome Biology. 2015;16(1):278.

11. Travaglini KJ, Nabhan AN, Penland L, Sinha R, Gillich A, Sit RV, et al. A molecular cell atlas of the human lung from single-cell RNA sequencing. Nature. 2020;587(7835):619-25.

12. Hao Y, Hao S, Andersen-Nissen E, Mauck WM, Zheng S, Butler A, et al. Integrated analysis of multimodal single-cell data. Cell. 2021;184(13):3573-87.e29.

13. Wu T, Hu E, Xu S, Chen M, Guo P, Dai Z, et al. clusterProfiler 4.0: A universal enrichment tool for interpreting omics data. Innov. 2021;2(3):100141.

14. Subramanian A, Tamayo P, Mootha VK, Mukherjee S, Ebert BL, Gillette MA, et al. Gene set enrichment analysis: A knowledge-based approach for interpreting genome-wide expression profiles. Proc National Acad Sci. 2005;102(43):15545-50.

15. Liberzon A, Subramanian A, Pinchback R, Thorvaldsdóttir H, Tamayo P, Mesirov JP. Molecular signatures database (MSigDB) 3.0. Bioinformatics. 2011;27(12):1739-40.

16. Liberzon A, Birger C, Thorvaldsdóttir H, Ghandi M, Mesirov Jill P, Tamayo P. The Molecular Signatures Database Hallmark Gene Set Collection. Cell Syst. 2015;1(6):417-25.

17. Kuri-Cervantes L, Pampena MB, Meng W, Rosenfeld AM, Ittner CAG, Weisman AR, et al. Comprehensive mapping of immune perturbations associated with severe COVID-19. Sci Immunol. 2020;5(49):eabd7114.
